# Forgotten signs of chronic kidney disease-associated mineral bone disease

**DOI:** 10.1093/qjmed/hcz211

**Published:** 2019-08-14

**Authors:** P G Hunter, E Miller-Hodges, R W Hunter, N Dhaun

**Affiliations:** Department of Renal Medicine, Royal Infirmary of Edinburgh, 53 Little France Crescent, Edinburgh EH16 4SA, UK

Chronic kidney disease (CKD) is a global health problem, estimated to affect over 500 million people worldwide.^1^ Over 80% of the world’s CKD population live in low- and middle-income countries (LMICs).^1^ Current data suggest that the prevalence of CKD is rising globally and the demographic transitions driving this trend (such as hypertension and diabetes) disproportionately affect LMIC populations.^2^ The healthcare infrastructure in many LMICs is ill-equipped to cope with this rising burden of CKD.^3^ As a consequence, access to and resources for preventative strategies and early CKD care models are limited and lack efficacy.^4^ Without service provision strategies for early diagnosis and treatment of CKD, progression to end-stage renal failure (ESRF) is inevitable. This presents further challenges in terms of access to renal replacement therapy, healthcare costs and mortality.

The rising burden of progressive CKD in LMICs will be associated with an inevitable rise in CKD-related complications such as secondary hyperparathyroidism and CKD-mineral bone disease (CKD-MBD). CKD-MBD is characterized by abnormalities of calcium and phosphate homeostasis, endocrine feedback, bone turnover and extra-skeletal calcification. The management of CKD-MBD was revolutionized in the 1970s following the introduction of activated vitamin D3 (1-hydroxycholecalciferol and 1,25-dihydroxycholecalciferol) into clinical practice. Extreme phenotypes of the skeletal and extra-skeletal complications of CKD-MBD are now rarely encountered in developed countries. Advanced manifestations of CKD-MBD, however, are likely to be commonplace in LMICs where diagnosis of advanced CKD is likely to be delayed and resources, however simple, limited to manage its complications.

**Figure 1.**
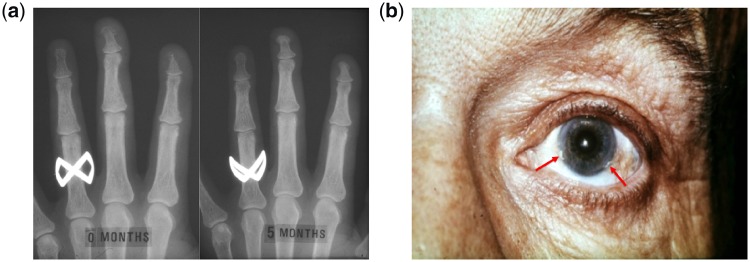
(**a**), Left Panel) Shows an X-ray of the left hand of a 27-year old female with ESRF taken immediately prior to her commencing activated vitamin D3 therapy. It demonstrates bone resorption of the terminal phalanges. (**a**, Right Panel) Shows the X-ray taken 5 months later and shows remarkable bone remodelling, secondary to activated vitamin D3 therapy, most evident at the terminal phalanx of the second finger. (**b**) Shows a photograph of the left eye of a 44-year old male with ESRF. It demonstrates calcification of the corneal limbus at the 5 and 8 o'clock positions.

Here, we present two historical cases from a tertiary renal unit in Edinburgh, Scotland, which exemplify the skeletal and extra-skeletal manifestations of CKD-MBD and highlight the efficacy of activated vitamin D3 as a treatment to control these sequelae. Figure 1a (left panel) shows an X-ray of the left hand of a 27-year old female with ESRF due to reflux nephropathy. The X-ray was taken immediately prior to her commencing activated vitamin D3 therapy and demonstrates bone resorption of the terminal phalanges. Figure 1a (right panel) shows the X-ray taken 5 months later and demonstrates remarkable bone remodelling, secondary to activated vitamin D3 therapy, most evident at the terminal phalanx of the second finger. Her plasma parathyroid hormone concentration fell from 4.1 to 1.2 µg/l over this period. The second case is of a 44-year old male with ESRF secondary to congenital renal dysplasia who started haemodialysis in 1964 at the age of 24. Figure 1b shows a photograph of his left eye with calcification of the corneal limbus demonstrated at the 5 and 8 o’clock positions.

Although activated vitamin D_3_ analogues are inexpensive [c. £60 ($80) per patient year], they are one of the most efficacious drugs at our disposal; a fact that is easy to take for granted in the 21st century.

*Conflict of interest*: none.
